# Congenital Malformations in the Moroccan Surveillance System: Contribution to Prevalence Estimation

**DOI:** 10.1155/2024/9570798

**Published:** 2024-03-18

**Authors:** Soukaina Kannane, Samia Boussaa, Jamila El Mendili, Oulaid Touloun

**Affiliations:** ^1^Polyvalent Team of Research and Development (PTRD), Polydisciplinary Faculty, Sultan Moulay Slimane University, 23000 Beni Mellal, Morocco; ^2^Higher Institute of Nursing and Health Techniques, Ministry of Health and Social Protection, 10000 Rabat, Morocco; ^3^Health Studies and Information Unit, Planning and Studies, Division, Planning and Financial Resources Department, Ministry of Health and Social Protection, 10020 Rabat, Morocco

## Abstract

**Background:**

Congenital malformations (CMs) are a group of structural or functional anomalies present at birth. These anomalies result in a high rate of mortality, morbidity, and disability in children. CMs are a major public health problem and place a heavy burden on healthcare systems in both developed and developing countries including Morocco, which has had a CMs surveillance system in place since 2011. The aim of this study is to determine the prevalence of CMs in Morocco.

**Methods:**

In this study, the epidemiology of CMs in Morocco was assessed retrospectively using the national surveillance system data gathered from case notification forms from 2017 to 2021.

**Results:**

The main results showed that the prevalence of CMs in Morocco is 3.91/1000 live births (LBs), and the minimum annual prevalence of CMs was reached in 2017 (3.10/1000 LBs) while the maximum annual prevalence was recorded in 2018 (4.55/1000 LBs). The majority of CMs are unspecified CMs (68.55%), neural tube defects (NTDs) account for (21.13%), and orofacial clefts (OFCs) account for (10.32%). In addition, the majority of CMs (61.73%) were from rural areas. According to region, the Dakhla-Oued Eddahab region recorded the highest prevalence of CMs in Morocco, with 8.81/1000 LBs, while the lowest prevalence was recorded in the Rabat-Sale-Kenitra region, with 2.02/1000 LB.

**Conclusions:**

This study reveals that the national prevalence of CMs is high and may be underestimated, as most of the CMS reported is unspecified. The use of a CM registry with detailed reporting of all CMs and the promotion of preventive measures are urgently recommended.

## 1. Context

Congenital malformations (CMs) are part of congenital anomalies, also known as birth defects, and are defined as a group of structural or functional abnormalities present at birth [1, 2] often classified by a system type according to the International Classification of Diseases ICD-10 [3]. These anomalies lead to a high rate of mortality and morbidity in children, with lifelong disability, having a direct impact on the newborn, parents, family, and healthcare systems. The World Health Organization (WHO) estimated that in 2017, CM was the cause of death of 283,582 newborns within 28 days of birth, as well as the death of 474,229 children under 5 years of age [4].

According to March of Dimes on birth defects [5], 8 million children are born worldwide with major congenital malformations every year, with the prevalence ranging from 47.2 in developed countries to 64.2 per 1,000 live births (LBs) in low-income countries [5]. The highest prevalence of CMs is in the Middle East and North Africa [5].

The etiology of CMs remains unknown in 50% of the cases [2]. Nevertheless, genetic factors, environmental teratogenic factors, and multifactorial causes are thought to be at the origin of some CMs [6]. Fortunately, the prevention of some CMs is still possible through the adoption of preventive measures [1, 7] such as a healthy diet, folic acid (FA) and iodine fortification and supplementation, rubella vaccination, prevention of certain infections such as syphilis and toxoplasmosis, preconception health care, and genetic counselling.

According to the Moroccan Ministry of Health, in 2020, CMs constitute a public health problem, representing the cause of death for 19.3% of children under the age of 5 [8] and 1.4% of the general population [8].

The national prevalence of CMs is not available to date despite the establishment of a CD surveillance system in 2011, whose reports are limited to neural tube defects (NTDs) and orofacial clefts (OFCs), without specifying the types of other CMs [9]. However, local studies concentrated in the capital Rabat have attempted to give the prevalence of CMs. Thus, Sabiri et al. [10] in 2013 found that CMs represented 4% of births and then in 2020, Forci et al. [11] found that the prevalence of these anomalies was 5.58/1000 births, while Elghanmi et al. [12] found the prevalence to be 10.2/1000 births in 2020.

A number of preventive measures have been introduced in Morocco, such as the fortification of flour with folic acid (FA) in 2005 and FA supplementation in 2008, as well as iodine fortification. Other preventive measures have been implemented, such as rubella vaccination and prevention of sexually transmitted infections; currently, screening for neonatal hypothyroidism and screening for congenital deafness have been implemented.

However, the impact of these measures on children's health can only be assessed by means of epidemiological indicators such as the prevalence of CMs.

In the absence of national CMs prevalence data, our study aims to determine the national epidemiological situation, providing a baseline for epidemiological surveillance of CMS. Consequently, the results will guide local program managers and international partner organizations in identifying gaps, implementing effective prevention strategies, and providing appropriate services.

## 2. Materials and Methods

### 2.1. Study Area

This study was carried out in Morocco, a country located in north-west Africa, with a surface area of 710,850 km^2^. In 2022, the Moroccan population was estimated at 36,670,216 and the national number of births in public establishments was estimated at 684,738 [8].

### 2.2. Data Collection

To calculate and determine the national prevalence of CMs in Morocco, a national study was carried out from 2017 to 2021 based on CMs epidemiological surveillance data from the Ministry of Health and Social Protection, whose national program for the prevention and control of micronutrient deficiencies ensures active surveillance of CMs in all the country's public health establishments. This surveillance is based on notification reports which are systematically transferred each month by the surveillance program coordinators after the collection of the notifications from the health personnel. The notification reports are transferred via an online application to the Planning and Financial Resources Department of the Ministry of Health and Social Protection, in particular to the Planning and Studies Division, which is responsible for data processing.

Diagnosis of clinically detectable CMs in newborns is made through a systemic examination conducted by healthcare providers such as midwives, pediatricians, general practitioners, and nurses. All cases were confirmed by pediatricians and/or general practitioners.

In order to calculate the prevalence of CMs, the research also focused on national births registered by region during the same period, also available from the Planning and Financial Resources Department of the Ministry of Health and Social Protection, in particular the Planning and Studies Division.

At the level of the CMs surveillance system, prior to 2017, notification was limited to NTDs and OFCs, so a lack of data from certain regions was observed. Since 2017, notification covered all clinically detectable CMs (minor and major), so data from all regions were available.

### 2.3. Ethical Considerations

The researchers were authorized by the Moroccan Ministry of Health and Social Protection to collect the data (Ref. no. 482/2021).

### 2.4. Data Analysis

Data were entered and analyzed using SPSS version 21.0 and Microsoft Excel. Descriptive analyses were used to describe the distribution of case characteristics.

Prevalence was calculated by dividing the number of newborns with congenital malformations by the total number of newborns living during the study period and included in the study (sample size) multiplied by 1000 [13].

## 3. Results

Among the 2,244,673 live births recorded in Morocco covering 5 years, between 2017 and 2021, a total of 8766 cases of CMs were recorded with a prevalence of 3.91/1000 LBs, with an average of 1753 cases per year, a variance of 63751.7 cases and a standard deviation of 252.49 cases.

**Figure 1:**
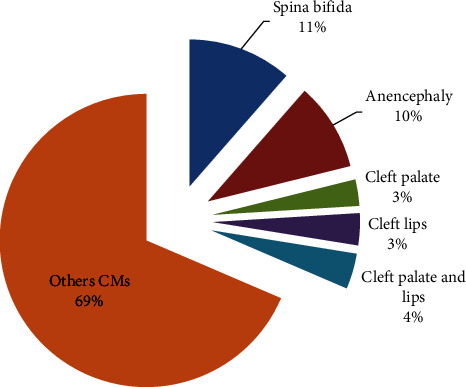
Distribution of CMs in Morocco between 2017 and 2021.

The breakdown by type of CMs concerned by notification in Morocco revealed that the majority (68.55%) are CMs without precision which encompass all clinically detectable CMs, while NTDs represent 21.13% (11.44% for spina bifida and 9.69% for anencephaly) and OFCs represent 10.32% (2.9% cleft palate (CP), 3.51% cleft lip (CL), and 3.91% cleft palate and lip (CPL)) (Figure 1).

**Figure 2:**
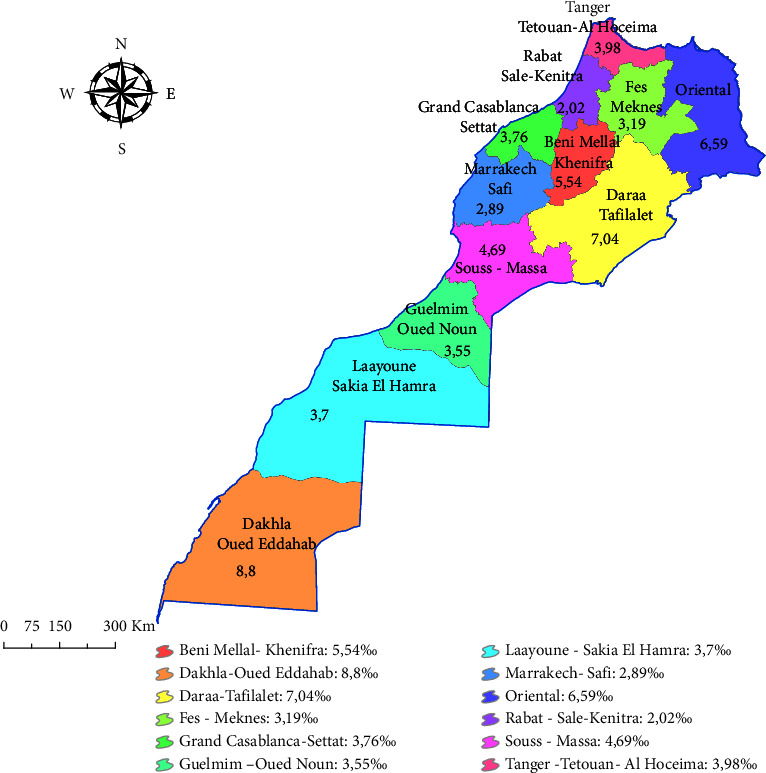
Prevalence of CMs in Morocco among administrative region from 2017 to 2021.

We note that the majority of CMs (61.73%) are located in rural areas while 38.27% are in urban areas. The overall prevalence of CMs varied between 2.02 and 8.81/1000 LBs depending on the region, with the Dakhla-Oued Eddahab region recording the highest prevalence of cases in Morocco at 8.81/1000 LB. The lowest prevalence was recorded in the Rabat-Sale-Kenitra region with 2.02/1000 LBs (Figure 2).

**Figure 3:**
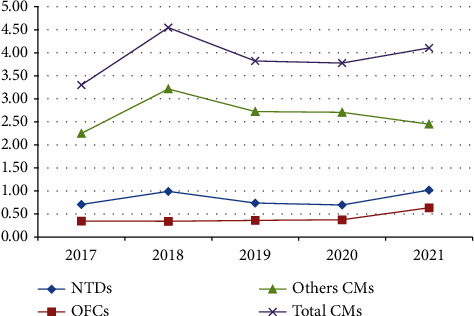
Annual prevalence of CMs detected by the national monitoring of CMs from 2017 to 2021.

The minimum overall prevalence of CMs was reached in 2017 (3.10/1000 LBs), followed directly by the maximum overall prevalence of cases in 2018 (4.55/1000 LBs), while the minimum number was recorded in 2017 (Figure 3).

In 2017, the annual prevalence of other CMs was minimal (3.10/1000 LBs), peaking in 2018 (4.55/1000 LBs), while a decrease took place between 2019 and 2020 (3.82 and 3.78/1000 LBs), followed by an increase in 2021 (4.10/1000 LBs).

**Figure 4:**
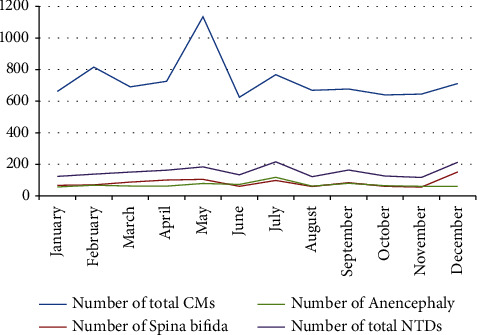
Distribution of the total number of total CMs and NTDs cases by month from 2017 to 2021 in Morocco.

Analysis of the temporal distribution of CMs (by month) showed that the maximum number of cases was reached in May, followed directly by the minimum number recorded in June. Other peaks were recorded in February, July, and December (Figure 4).

The national prevalence of NTDs is 0.83/1000 LBs (0.45/1000 LBs for spina bifida and 0.38/1000 LBs for anencephaly), the highest being recorded in the Oriental region with 1.77/1000 LBs and the lowest in the Rabat-Sale-Kenitra region with 0.44/1000 LBs. The highest prevalence was recorded in the Oriental region at 1.77/1000 LBs, and the lowest in the Rabat - Sale-Kenitra region at 0.44/1000 LBs (Table 1).

The minimum prevalence of all NTDs was reached in 2017 (0.71/1000 LBs), while the maximum prevalence was recorded in 2021 (1.02/1000 LBs). Another peak was recorded in 2018 (0.99/1000 LBs), followed by a decrease in 2019 and 2020 (Figure 3). The maximum number of NTDs cases was reached in July, with another peak in December. The lowest number of cases was reached in January, August, and November. The same pattern was observed for spina bifida cases. As for cases of anencephaly, the highest number of cases was also recorded in July (Figure 4).

The national prevalence of OFCs is 0.40/1000 LBs (0.11/1000 LBs for CP, 0.14/1000 LBs for CL, and 0.15/1000 LBs for CPL) (Table 2). The highest prevalence of OFCs is recorded in the Dakhla-Oued Eddahab region with 1/1000 LBs, and the lowest prevalence of OFCs is recorded in the Rabat-Sale-Kenitra region with 0.18/1000 LBs (Table 2).

**Figure 5:**
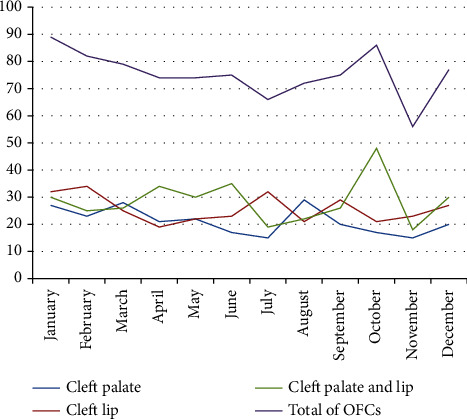
Distribution of type of OFCs cases by month from 2017 to 2021 in Morocco.

With regards to the prevalence of OFCs, a slight increase was observed between 2018 and 2020, with 0.01 cases per 1000 LBs per year, followed by a peak recorded in 2021 (0.64/1000 LBs) (Figure 3). It is worth noting that the number of OFCs gradually declined over the first six months of the year, gradually increasing until reaching a peak in October, with a significant drop the following month. The same observation was made for CPL cases. For CP, a peak was reached in August, followed by a minimum number of cases in July. The number of CL cases was high in January and February, followed by a decrease and quasistability the rest of the time (Figure 5).

## 4. Discussion

The Millennium Development Goals (Goal 4) and Sustainable Development Goals (Goal 3) include reducing child mortality and ensuring good health and well-being [14]. These objectives cannot be achieved without thinking about CMs, which represent a serious public health problem on a global scale. Internationally, these anomalies were the fourth leading cause of death in children under 5 in 2019, contributing to around 10% of all deaths [15].

In Africa, the percentage of all under-5 mortality attributable to CMs rose from 4.6% in 2000 to 7.6% in 2019. [16]. Thus, CMs significantly reduces survival and causes various types of disability [17]. In addition, both major and minor CMs have been associated with severe maternal morbidity, such as caesarean delivery, chorioamnionitis, and postpartum endometritis [18]. In addition, these embryopathies also affect the psychological health of parents, generating high levels of stress, anxiety, and depression [19].

As reported by March of Dimes on birth defects [5] worldwide, 8 million children are born each year with major CMs. The prevalence of CMs depends on the level of national income. As a result, the prevalence is lower in high-income countries than in low- and middle-income countries; in low-income countries, the prevalence of CMs is 64.2/1000 LBs, in developing countries, 55.7/1000LBs, and in developed countries, 47.2/1000LBs [5]. The highest prevalence of CMs is found in the Middle East and North Africa, more specifically in Sudan with 82/1000LBs [5].

To date, the causes of the majority of CMs are unknown [2]. However, genetic factors, environmental teratogenic factors, and multifactorial factors are thought to be at the origin of some CMs [6]. Fortunately, some CMs can be prevented by adopting preventive measures [1, 7, 20].

In Morocco, 19.3% of under-5 mortality were attributable to CMs in 2016 [8]. In addition, according to the results of the second national disability survey [21] done in 2014, the national prevalence of disability is 6.8%, which means that CMs are a public health problem.

Some CMs prevention measures have been adopted in Morocco, such as folic acid fortification of flour in 2005 and folic acid supplementation in 2008, while other prevention measures have been implemented, such as rubella vaccination and prevention of sexually transmitted infections. Furthermore, screening for neonatal hypothyroidism and screening for congenital deafness have recently been implemented. The impact of the various interventions on birth outcomes should be realized by evaluating a CMs surveillance system [9].

In Morocco, despite the introduction of a surveillance system for CMs in 2011 [9] the national prevalence of CMs is not yet available. However, there are a few local studies targeting the prevalence of CMs, such as the study carried out in Rabat in 2020 [11] which estimated the prevalence of these anomalies at 5.58/1000 births and another study carried out in the same context [12] found a prevalence of 10.2/1000 births. In Rabat, CMs accounted for 4% of births in 2010 [10].

It should be noted that the surveillance system for CMs in Morocco has been in place since 2011 as part of the monitoring and evaluation system for the national iron and folic acid flour fortification program. In addition, reporting was limited to neural tube defects (NTDs) and orofacial clefts (OFCs) [9]. The monitoring was extended to include other CMs only in 2017. Medical records and maternal interviews were the source of information for active notification, carried out on a monthly basis and covering children of all ages from birth onwards, and including both stillbirths and terminations [9]. National surveillance of CMs is not based on the use of the CMs registry, with the exception of a single center in the capital Rabat; yet, the registry is considered the most effective tool for surveillance, research, and evaluation of public health interventions [22].

This is the first situational analysis carried out in Morocco concerning CMs; the results of this study revealed that the national prevalence of CMs is 3.91/1000LBs, which is close to the prevalence found in local studies carried out between 2011 and 2016 in the capital Rabat in 2020 [23] estimating CMs prevalence at 5.58/1000 births, while another study carried out between 2011 and 2014 in the same context [12] found a higher prevalence than our result of 10.2/1000 births.

A review of sub-Saharan African countries published in 2020 [24] revealed that the pooled prevalence of CMs was 20.40/1000 births, while in Europe, the estimated prevalence of CMs was 25.0/1000 births [25]. Worldwide, around 3% of children suffer from major CMs [22].

It should be noted that the national prevalence of total CMs reported in our research is very low compared with other prevalence recorded in Africa and Europe. The prevalence of total CMs found is much lower than the prevalence estimated by Mach of Dimes for Morocco (72.7/1000 LBs) [26] indicating a huge underestimation. This underestimation may result from a deficiency in the surveillance system, which does not include birth defects of genetic or partially genetic origin accounting for about 25% of all of CMs [5], national notification concerns only clinically detectable CMs so that invisible anomalies and functional disorders are not diagnosed and, therefore, not taken into account. In addition, notification does not include CMs in case of abortions [9]. Indeed, among the clinically detectable CMs identified by the Moroccan surveillance system, there are no details of all CMs with the exception of OFCs (three types…) and NTDs (only spina bifida and anencephaly). In addition, the reporting system does not cover children born and cared for in the private sector.

During the present study, a significant disparity was observed between rural and urban areas (61.73% of CMs were recorded in rural areas). However, according to the latest population census in Morocco, the urbanization rate is 60.3% [27]. The results of this study suggest that in rural areas, the high prevalence of CMs may be explained by precarious living conditions, poverty, lack of hygiene and medical follow-up before and during pregnancy, the use of pesticides in agriculture, and the fact that in rural areas, the total fertility rate is 2.4 children/woman, whereas in urban areas in Morocco, it is 1.9 children/woman [8].

It should be noted that a significant regional disparity was recorded. On the one hand, the highest prevalence of all CMs as well as OFCs is recorded in the Dakhla-Oued Eddahab region (8.81/1000 LBs and 1/1000 LBs respectively), which can be associated to the highest smoking rate in Morocco (16.4%) recorded in this region [27]. However, the highest prevalence of NTDs is recorded in the Oriental region (1.77/1000 LBs). On the other hand, the lowest overall prevalence of CMs, NTDs, and OFCs are reported in the Rabat-Sale-Kenitra region, which can be justified by favorable health conditions, such as the high urbanization rate (72.2%), the low unemployment rate (12.2%) in 2021, and the fact that more than half of Morocco's practicing physicians are concentrated in the Rabat-Sale-Kenitra and Greater Casablanca-Settat regions [27].

The national prevalence of NTDs found in our study is 0.83/1000 LBs, which is lower than the estimated prevalence of NTDs for Morocco reported by Kancherla [28] (1.75/1000 LBs); also, this prevalence is notably lower than the Mach of Dime estimation [26] (2.2/1000 LBs). Our prevalence is double the prevalence found [29] in a multicenter study (20 public hospitals in Morocco) with a duration of three years (2012–2014), i.e., a prevalence of 0.43/1000 LBs. However, this multicenter study excluded stillbirths, fetal deaths in utero after 22 weeks' amenorrhea, and medical terminations of pregnancy. Other local Moroccan studies have shown that the annual prevalence of NTDs [30] has decreased significantly, from 2.17 in 2008 to 1.21/1000 births in 2011. In 2021, another Moroccan study [31] showed that the prevalence rate of NTDs was 1 per 1000 LBs in Rabat. It should be noted that the national prevalence of NTDs detected in our study is lower than the worldwide prevalence of NTDs, which is 1.86/1000 LBs [28].

According to our results, the prevalence of spina bifida and anencephaly was 0.45 and 0.38/1000 LBs, respectively, whereas Laamri [29] concluded in a study of NTDs that the prevalence from 2012 to 2014 of spina bifida and anencephaly was 0.19 and 0.23/1000 LBs, respectively.

With regards to OFCs, the national prevalence in our study (0.40/1000 LBs) is double the prevalence found in the Moroccan multicenter study by Laamiri [29] which was 0.25/1000 LBs. Our results are lower than the pooled prevalence of OFCs in low- and middle-income countries, which was approximately 1.38/1000 births [32]. Internationally, a recent systematic review published in 2023 concluded that the global prevalence of OFCs was estimated at 4.6 million children [29].

According to a meta-analysis published in 2021 [33], the worldwide prevalence of cleft palate, cleft lip, and cleft palate was 0.33, 0.30, and 0.45/1000 LBs, respectively. In contrast, our results showed that the national prevalence of cleft palate, cleft lip, and cleft lip/palate was 0.11, 0.14, and 0.15/1000 LBs, respectively.

No seasonal variation was detected in our analysis of OFC cases, whereas in South Africa, a study [34] showed seasonal variation only for patients presenting with both FFOs. Another Chinese study [35] showed a seasonal variation in OFC patients, with peak prevalence at birth during winter. In contrast, a trend towards seasonality was observed in spring in Brazil between 2008 and 2019 [36].

Another Chinese study [35] found a seasonal variation in people with OFCs, with peak prevalence at birth during winter. In contrast, a trend towards seasonality was observed in spring in Brazil between 2008 and 2019 [36].

In our context, there are many risk factors for the onset of CMs, such as 30.3% of women suffering from anemia [27], 29% of Moroccan women are obese [27], and 51.3% of women are illiterate [27]. Moreover, there is a lack of knowledge about the risk of consuming teratogenic plants such as fenugreek, which has been associated in Moroccan studies [10, 37, 38] to the occurrence of CMs, particularly NTDs, and the same is true for the use of antiepileptic drugs [10]. Fortunately, only 1% of Moroccan women are smokers [27].

Similarly, the average age of marriage is high in Morocco, at 25.5 for women [27]. Consanguineous marriages are more frequent in Morocco. A Moroccan study [39] showed that consanguinity is a risk factor for CMs.

A recent study [31] conducted in the Moroccan capital revealed that none of the mothers of newborns with NTDs took FA supplements before conception, and only 41% of them did so during the first trimester of pregnancy.

On the one hand, certain limitations need to be taken into account when interpreting the results, notably the fact that unfortunately, the national surveillance system focuses on NTDs and OFCs notification without giving details about other CMs. Besides, CMs are not notifiables diseases and, therefore, omissions from notification are possible and also that private sector data on CMs are not available.

On the other hand, the Ministry of Health and Social Protection has recently introduced the use of computerized medical records in both the public and private sectors, enabling more effective national monitoring of CMs.

Based on our findings, it is recommended to generalize the use of the CMs registry for the whole kingdom to include all CMs in detail, in particular to establish a network of registries that plays a key role in coordinating CMs surveillance and research in Morocco. It is also recommended to increase healthcare provision by ensuring a sufficient number of genetic study centers to cover the surveillance of genetic abnormalities.

## 5. Conclusions

CMs remain a major cause of mortality, infant morbidity, and disability in Morocco. This study presents the first situational analysis carried out in Morocco concerning these embryopathies, revealing that national prevalence is significantly underestimated. Health system decision-makers need to generalize the use of the CMs registry for more effective epidemiological surveillance, insist on screening and detailed notification of all CMs, raise awareness among healthcare professionals about the importance of notification, and emphasize the necessity of preventive measures such as folic acid supplementation before and during pregnancy.

## Figures and Tables

**Table 1:** Prevalence per 1000 LBs of NTDs by type in Morocco from 2017 to 2021.

Regions	Total number of birth live from 2017 to 2021	Number of NTDs	Prevalence of NTDs	Number of spina bifida	Prevalence of spina bifida	Number of anencephaly	Prevalence of anencephaly
Tanger-Tetouan-Al Hoceima	243443	226	0.93	107	0.44	119	0.49
Oriental	130639	231	1.77	179	1.37	52	0.40
Fes-Meknes	298220	240	0.80	119	0.40	121	0.41
Rabat-Sale-Kenitra	313130	139	0.44	73	0.23	66	0.21
Beni Mellal-Khenifra	163842	222	1.35	130	0.79	92	0.56
Grand Casablanca-Settat	370040	169	0.46	104	0.28	65	0.18
Marrakech-Safi	346092	201	0.58	113	0.33	88	0.25
Daraa-Tafilalet	123776	156	1.26	67	0.54	89	0.72
Souss-Massa	175294	206	1.18	87	0.50	119	0.68
Guelmim-Oued Noun	33748	27	0.80	11	0.33	16	0.47
Laayoune-Sakia El Hamra	33502	29	0.87	10	0.30	19	0.57
Dakhla-Oued Eddahab	12947	6	0.46	3	0.23	3	0.23
Total	2244673	1852	0.83	1003	0.45	849	0.38

**Table 2:** Prevalence per 1000 LBs of OFCs by type in Morocco from 2017 to 2021.

Regions	Number of birth live	Total of OFCs	Prevalence of OFCs	Number of cleft palate	Prevalence of cleft palate	Number of cleft lip	Prevalence of clefts lip	Number of cleft palate and lip	Prevalence of cleft palate and lip
Tanger-Tetouan-Al Hoceima	243443	93	0.38	34	0.14	26	0.11	33	0.14
Oriental	130639	99	0.76	35	0.27	32	0.24	32	0.24
Fes-Meknes	298220	99	0.33	44	0.15	30	0.10	25	0.08
Rabat-Sale-Kenitra	313130	57	0.18	23	0.07	8	0.03	26	0.08
Beni Mellal-Khenifra	163842	92	0.56	14	0.09	30	0.18	48	0.29
Grand Casablanca-Settat	370040	127	0.34	23	0.06	57	0.15	47	0.13
Marrakech-Safi	346092	131	0.38	31	0.09	46	0.13	54	0.16
Daraa-Tafilalet	123776	80	0.65	19	0.15	27	0.22	34	0.27
Souss-Massa	175294	77	0.44	18	0.10	33	0.19	26	0.15
Guelmim-Oued Noun	33748	17	0.50	2	0.06	9	0.27	6	0.18
Laayoune-Sakia El Hamra	33502	20	0.60	4	0.12	5	0.15	11	0.33
Dakhla-Oued Eddahab	12947	13	1.00	7	0.54	5	0.39	1	0.08
Total	2244673	905	0.40	254	0.11	308	0.14	343	0.15

## Data Availability

The epidemiologic data used to support the findings of this study are available from the corresponding author upon request.

## Abbreviations

B: Birth
CL: Cleft lip
CLP: Cleft lip and palate
CP: Cleft palate
CMs: Congenital malformation
FA: Folic acid
LB: Live birth
NTDs: Neural tube defects
OFCs: Oro-facial clefts.

## References

[1] DeSilva M., Munoz F. M., Mcmillan M. (2016). Congenital anomalies: case definition and guidelines for data collection, analysis, and presentation of immunization safety data. *Vaccine*.

[2] World Health Organization (2023). Principaux repères sur les anomalies. https://www.who.int/fr/news-room/fact-sheets/detail/birth-defects.

[3] Agence technique de l’information sur l’hospitalisation (2017). Classification statistique internationale des maladies et des problèmes de santé connexes. https://www.atih.sante.fr/sites/default/files/public/content/3069/cim-10_fr_2017.pdf.

[4] World Health Organization (2023). Number of deaths in children aged <5 years, by. https://www.who.int/data/gho/data/indicators/indicator-details/GHO/number-of-deaths.

[5] March of Dimes (2006). Global report on birth defec T S. https://www.prevencioncongenitas.org/wp-content/uploads/2017/02/Global-report-on-birth-defects-The-hidden-toll-of-dying-and-disabled-children-Full-report.pdf.

[6] Lee K. S., Choi Y. J., Cho J. (2021). Environmental and genetic risk factors of congenital anomalies: an umbrella review of systematic reviews and meta-analyses. *Journal of Korean Medical Science*.

[7] Kancherla V., Oakley G. P., Brent R. L. (2014). Urgent global opportunities to prevent birth defects. *Seminars in Fetal and Neonatal Medicine*.

[8] WHO (2021). Ministry of health and social protection in Morocco D of P and F resources. *Health in numbers 2020*.

[9] Yunis K., Al Bizri A., Al Raiby J. (2021). Situational analysis of the surveillance of birth defects in the Eastern Mediterranean region. *International Journal of Epidemiology*.

[10] Sabiri N., Kabiri M., Razine R., Kharbach A., Berrada R., Barkat A. (2013). Facteurs de risque des malformations congénitales: étude prospective à la maternité Souissi de Rabat au Maroc. *Journal de Pediatrie et de Puericulture*.

[11] Forci K., Alami M. H., Bouaiti E., Slaoui M., Mdaghri Alaoui A., Thimou Izgua A. (2020). Prevalence of congenital malformations at the “les Orangers” maternity and reproductive health Hospital of Rabat: descriptive study of 470 anomalies. *BMC Pediatrics*.

[12] Elghanmi A., Razine R., Jou M., Berrada R. (2020). Congenital malformations among newborns in Morocco: a retrospective study. *Pediatric Reports*.

[13] Sbai-Idrissi K., Galoisy-Guibal L., Boutin J. P. (2002). Que sont l’incidence et la prevalence?. *Medecine Tropicale: revue du Corps de sante colonial*.

[14] Yunus F. M., Khan S., Khanam F., Das A., Rahman M. (2019). Summarizing the recommendation of arsenic research during Millennium development Goals (MDGs) era in Bangladesh-future directions for the sustainable development Goals (SDGs). *Groundwater for Sustainable Development*.

[15] Kang L., Cao G., Jing W., Liu J., Liu M. (2023). Global, regional, and national incidence and mortality of congenital birth defects from 1990 to 2019. *European Journal of Pediatrics*.

[16] Perin J., Mai C. T., De Costa A. (2023). Systematic estimates of the global, regional and national under-5 mortality burden attributable to birth defects in 2000-2019: a summary of findings from the 2020 WHO estimates. *BMJ Open*.

[17] Malherbe H. L., Aldous C., Christianson A. L., Darlison M. W., Modell B. (2021). Modelled epidemiological data for selected congenital disorders in South Africa. *Journal of Community Genetics*.

[18] Kawakita T., Gustavo V. I. L. C. H. E. Z., Lea N., Huang J. C., Molly H. O. U. S. E. R., Jose Duncan M. A. Adverse maternal outcomes associated with major fetal malformations after singleton live birth. *American Journal of Obstetrics and Gynecology MFM*.

[19] Al-Maghaireh D. F., Kawafha M. M., Abdullah K. L., Shawish N. S., Abu Kamel A. M., Basyouni N. R. (2023). Psychological problems among parents of children with congenital anomalies. *Journal of Neonatal Nursing*.

[20] Taruscio D., Bermejo-Sánchez E., Paolo Salerno A. M. (2019). Primary prevention as an essential factor ensuring sustainability of health systems: the example of congenital anomalies. *Annali dell’Istituto Superiore di Sanita*.

[21] de la Solidarité M., De la Femme D. S. (2014). Enquête nationale sur le Handicap 2014 Rapport détaillé. https://social.gov.ma/wp-content/uploads/2024/03/2.

[22] Khoshnood B., Lelong N., Kassis M. (2013). Registres de malformations congénitales: un outil pour la surveillance, la recherche et l ’ évaluation des actions de santé. *Bulletin de l’Académie Nationale de Médecine*.

[23] Forci K., Alami M. H., Bouaiti E., Slaoui M., Alaoui A. M., Izgua A. T. (2020). Prevalence of congenital malformations at the“les Orangers”maternity and reproductive health Hospital of Rabat: descriptive study of 470 anomalies. *BMC Pediatrics*.

[24] Adane F., Afework M., Seyoum G., Gebrie A. (2020). Prevalence and associated factors of birth defects among newborns in sub-saharan african countries: a systematic review and meta-analysis. *The Pan African medical journal*.

[25] Kinsner-Ovaskainen, Garne E. (2009). European monitoring of congenital anomalies JRC-EUROCAT report on statistical monitoring of congenital anomalies. https://ec.europa.eu/jrc.

[26] Lucia S., Salvador E., Rica C. (2001). *March of dimes*.

[27] Haut Comissariat au Plan (2022). *Les indicateurs sociaux du Maroc*.

[28] Kancherla V., Chadha M., Rowe L., Thompson A., Jain S., Walters D. (2021). Low-and middle-income countries: an analysis to identify large-scale mandatory fortification of wheat flour and rice. *Nutrients*.

[29] Laamiri F. Z. (2017). PREVALENCE of neural tube closure defects and the ORO-FACIAL. *World Journal of Pharmaceutical Research*.

[30] Radouani M. A., Chahid N., Benmiloud L. (2015). Prevalence of neural tube defects: Moroccan study 2008-2011. *Open Journal of Pediatrics*.

[31] Forci K., Bouaiti E. A., Alami M. H., Mdaghri Alaoui A., Thimou Izgua A. (2021). Incidence of neural tube defects and their risk factors within a cohort of Moroccan newborn infants. *BMC Pediatrics*.

[32] Kadir A., Mossey P. A., Orth M. (2017). Systematic review and meta-analysis of the birth prevalence of orofacial clefts in low- and middle-income countries. *The Cleft Palate-Craniofacial Journal*.

[33] Salari N., Darvishi N., Heydari M., Bokaee S., Darvishi F., Mohammadi M. (2022). Global prevalence of cleft palate, cleft lip and cleft palate and lip: a comprehensive systematic review and meta-analysis. *Journal of Stomatology, Oral and Maxillofacial Surgery*.

[34] Sofianos C., Christofides E. A., Phiri S. E. (2018). Seasonal variation of orofacial clefts. *Journal of Craniofacial Surgery*.

[35] Hao Y., Zhuang D., Jiao X. (2022). Seasonal variation of nonsyndromic orofacial clefts in northern Chinese population. *Journal of Craniofacial Surgery*.

[36] Calderon M. G., Oliveira Simoni V. C., de Sousa Santos E. F. (2023). Epidemiologic characteristics, time trend, and seasonality of orofacial clefts. *The Cleft Palate-Craniofacial Journal*.

[37] Skalli S. (2006). Malformations associées à la prise de fenugrec au cours de la grossesse Entre. *Bulletin d’information de pharmacovigilance*.

[38] Taloubi L. M., Rhouda H., Belahcen A., Smires N., Thimou A., Mdaghri A. A. (2013). An overview of plants causing teratogenicity: fenugreek (Trigonella foenum graecum). *International Journal of Pharmaceutical Sciences and Research*.

[39] Karim B., Saïd E. L. M. Congenital Anomalies in Neonates and Associated Risk Factors in Agadir Region of Morocco.

